# Bio-Inspired Distributed Transmission Power Control Considering QoS Fairness in Wireless Body Area Sensor Networks

**DOI:** 10.3390/s17102344

**Published:** 2017-10-14

**Authors:** Chan-Jae Lee, Ji-Young Jung, Jung-Ryun Lee

**Affiliations:** School of Electrical and Electronics Engineering, Chung-Ang University, 84 Heukseok-ro, Dongjak-gu, Seoul 06974, Korea; cjleeno22@cau.ac.kr (C.-J.L.); jiyoung@cau.ac.kr (J.-Y.J.)

**Keywords:** Bio-inspired, energy efficiency, fairness, power control, quality of service, wireless body area sensor network

## Abstract

Recently, the development of wireless body area sensor network (WBASN) has accelerated due to the rapid development of wireless technology. In the WBASN environment, many WBASNs coexist where communication ranges overlap with each other, resulting in the possibility of interference. Although nodes in a WBASN typically operate at a low power level, to avoid adversely affecting the human body, high transmission rates may be required to support some applications. In addition to this, since many varieties of applications exist in the WBASN environment, each prospective user may have different quality of service (QoS) requirements. Hence, the following issues should be considered in the WBASN environment: (1) interference between adjacent WBASNs, which influences the performance of a specific system, and (2) the degree of satisfaction on the QoS of each user, i.e., the required QoS such as user throughput should be considered to ensure that all users in the network are provided with a fair QoS satisfaction. Thus, in this paper, we propose a transmission power adjustment algorithm that addresses interference problems and guarantees QoS fairness between users. First, we use a new utility function to measure the degree of the satisfaction on the QoS for each user. Then, the transmission power of each sensor node is calculated using the Cucker–Smale model, and the QoS satisfaction of each user is synchronized dispersively. The results of simulations show that the proposed algorithm performs better than existing algorithms, with respect to QoS fairness and energy efficiency.

## 1. Introduction

With the rapid advance of wireless technology in recent years, the development of WBASN has been accelerated in an effort to monitor patients and support applications used for telemedicine [1,2,3]. As the population is growing rapidly around the world, the demand for healthcare systems will consequently increase. Especially the population aged between 60 and 80 is expected to double in 2050, compared to the number in 2010. Thus, technological development in WBASNs is essential. Millions of people die every year from diseases such as cancer and cardiovascular disease, where early detection and treatment can dramatically reduce the mortality rate. Therefore, future healthcare systems should provide preventive services. Wearable devices that can be attached to the body, to monitor and analyze a bio-signal, are typically used as effective precautionary measures, for early disease detection. Many studies have been conducted to address various problems that may occur in the WBASN environment, where these wearable devices are used.

The WBASN can be configured by several wireless technologies that have advantages and disadvantages depending on each radio technology [4]. In this paper, we focus on the IEEE 802.15.6 standard. A study group on the WBASN communication standard was approved as Task Group 6 (TG6) of the IEEE 802.15 working group, in January 2008. The first draft of the WBASN standard was ratified in February 2012 [5]. The purpose of WBASN communication standardization is “to provide an international standard for a short-range (i.e., about human body range), low power, and highly reliable wireless communication for use in close proximity to, or inside, a human body” [1].

The WBASN has distinctive features compared with the existing wireless networks [6]. In the WBASN environment, there are many WBASN networks where communication ranges overlap with each other. Thus, interference may occur between each WBASN network, because of limited frequency resources [7]. This inter-WBASN interference may cause problems that affect each user, such as a decrease in the throughput, or an increase in the packet loss rate. In general, inter-WBASN interference is maximized when there is no coordination between each network. Generally speaking, techniques for reducing interference can be classified as centralized and distributed methods. With centralized methods, a central coordinator is deployed like a base station, which regulates the media access control (MAC) and transmit power of each sensor node that is dependent on a WBASN, to reduce the effect of interference on each hub. However, since each user in the WBASN environment is highly mobile, and user-dependent WBASNs operate independently of each other, central coordinator based interference mitigation methods are not suitable for deployment with WBASNs. Therefore, distributed methods are used to mitigate interference in the WBASN environment.

**Figure 1:**
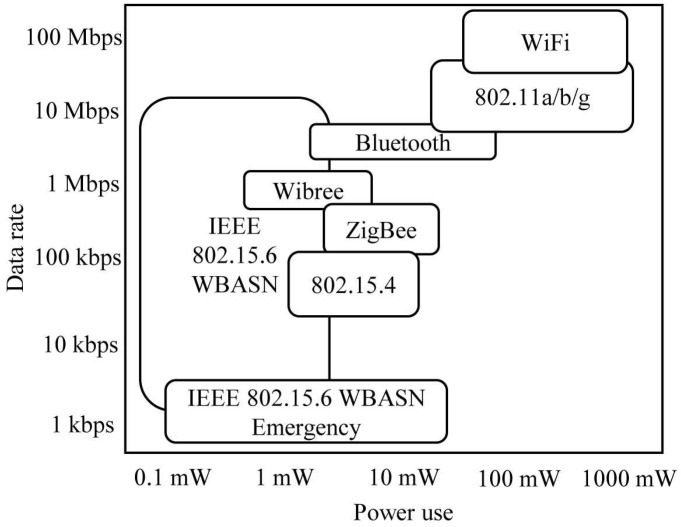
Comparison of power requirements and data rates in different wireless technologies.

Figure 1 shows the transmission power requirements and data rates for different wireless technologies [1]. From this image, it can be seen that the transmit power required in a WBASN environment is significantly lower than with other networks. Since the sensor nodes in the WBASN, for collecting biometric data, may exist on the inside, and on the surface of a human body, lower power consumptions are required for WBASN technology than with existing wireless technologies, in consideration of the specific absorption rate (SAR) of electromagnetic waves. In spite of the limited transmit power of WBASNs, the range of transmission rates required for each application is very broad. This is because the types of data measured by the sensor nodes vary depending on the application, and the required data rate varies depending on the type of data being transmitted. Since a maximum data rate of about 10 Mbps and low power consumption are required in the WBASN environment, the WBASN protocol requires higher energy efficiency than existing protocols. Depending on the type of application being used in a WBASN, the differences in the required data rate may be very large. As a result, some sensor nodes may use an unnecessarily large transmission power, reducing their energy efficiency, as well as the performance of other nodes. In addition, since the types of applications utilized in a WBASN are different, the data rate required for satisfying quality of service (QoS) may also be different for each WBASN. A required transmission rate based on the QoS is directly related to fair treatment of users in the network [8]. If the difference in required data rate for a satisfactory QoS is not taken into consideration, users in the network could be provided with an unfair service. For example, allocating the same amount of resources to a voice service of relatively low quality, and a high-quality video service, does not provide each user with fair treatment. Therefore, in the WBASN environment it is necessary to consider different QoS requirements according to the applications employed in WBASNs.

The remainder of this paper is organized as follows. In Section 2, we present the related works. In Section 3, we describe the general system models and parameters of the WBASN. In Section 4, we explain the Cucker–Smale model, which is the basis of the proposed algorithm. In Section 5, we introduce the operation of the proposed transmission power control algorithm in WBASN. In Section 6, we evaluate the performance of the proposed algorithm. Finally, we conclude this paper in Section 7.

## 2. Related Works

The interference problem has been dealt with in the existing network [9]. It is important to design the wireless network by considering the signal-to-interference-ratio (SINR), as indicated by the wide literature available on the topic, which we review in this section. The obtained SINR is proportional to a variety of performance metrics including throughput [10]. However, the frequencies assigned to two connections may incur interference to one another, resulting in quality loss of the signal [11]. For this reasons, several studies in the traditional wireless networks had conducted to mitigate interference. Resource allocation schemes in wireless network have been proposed to assign channel and to associate the users with access points (APs) by considering SINR. To address the co-channel interference problems, a biological behavior-based network resource management method is proposed [12]. A solution to user association with the AP is proposed to guarantee the best quality of service considering SINR [13]. In the cellular networks or the broadcasting systems, the efficiency of SINR is an important factor and the optimization of the SINR problem have been conducted. The planning methodologies in the cellular network that allow to minimize interference overhead while maintaining the established network coverage are introduced [14]. A solution method for SINR constraints problem in the cellular network is proposed that combines combinatorial Benders decomposition, classical Benders decomposition, and valid cuts in a nested way [15]. A two-stage heuristic methodology composed of power and frequency assignment stages is proposed so as to minimize the loss from mutual interference in the broadcasting system [16]. An optimization problem of transmission powers so as to guarantee the required SINR in the broadcasting system can be formulated as a mixed integer linear program, and the analysis on the behavior of this model is proposed [17]. To solve the interference problem caused by the coexistence of a plurality of WBASNs that are dependent on a specific individual, unlike earlier works for the existing wireless networks, many studies considering features of the WBASN have been conducted. The asynchronous inter-network interference avoidance (AIIA) scheme has been proposed, to reduce the magnitude of mutual interference that occurs in situations where two or more WBASNs share the same channel, so that the active interval in each WBASN frame is not allocated at the same time [18]. AIIA is based on a hybrid technique of carrier sense multiple access with collision avoidance (CSMA/CA) and time division multiple access (TDMA). In this scheme, information relating to time occupied in a TDMA interval is periodically exchanged and recorded in the AIIA table. The coordinator in each WBASN can check if its TDMA interval conflicts with the time occupied by a neighbor, based on the information in the AIIA table. If a collision is predicted, the coordinator occupies another time slot. A technique for adjusting the modulation parameter, data rate, and duty cycle, linearly, according to the SINR of the receiver has been proposed, to solve co-channel interference [19].

Transmission power control plays an important role in mitigating interference in a wireless network. The coverage extension of base stations (BSs) can be enhanced by mitigating interference in the cellular networks [20]. In this scheme, the optimization of BS parameters leads to improving the system performance. The overall power consumption of AP is also decreased by interference management in the wireless local area network (WLAN) [21]. This approach adapts the branch-and-benders-cut method to solve the non-linear power design problem on the interference management. Many studies have been conducted on methods to improve performance in the WBASN environment by adjusting transmission power. The received signal strength indicator (RSSI) is one of the commonly used parameters for power control algorithm. Due to inter-sensor node interference caused by body movement or variance of channel status, a method for detecting changes to the link quality using the received signal strength indicator (RSSI), and adjusting the transmission power, is proposed [22]. An energy efficient transmission power control scheme in an on-demand way to adapt to varying channel environments is proposed [23]. A power control schemes based on the reinforcement learning (RL) and mathematical optimization are proposed to consider interference. To determine the optimal combination of beamforming and power control in sensor arrays, the RL algorithm is proposed [24]. In this case, the power configuration set of this kind of scheme could be explored by [25]. A method for channel and power allocation based on a RL mechanism and convex optimization is proposed [26]. Studies on game theory-based algorithm have been conducted in existing wireless networks to obtain the optimal transmission power that maximizes system performance. The non-cooperative differential game is used to control transmit power of wireless powered sensor network [27]. This scheme extends working hours and improves throughput by optimal power control. The proactive power update (PAPU) algorithm applied this theory to WBASN environments with the purpose of maximizing the data rate of the entire WBASN networks [7]. The scheme recognizes changes in SINR or transmission power of neighboring WBASNs, and determines whether to adjust the transmission power. If a coordinator decides to adjust transmission power, the optimal power that increases the transmission rate is found using the Nash equilibrium, based on the quality of the channel, interference from other WBASNs, noise, and other predetermined power parameters.

Besides, studies on various problem occurring in WBASN have been conducted. Researches on using relays have conducted to improve performance of far nodes. In the system of using relay node, routing is essential to improve network lifetime and system performance. A global routing scheme using Dijkstra’s algorithm is proposed with a novel cost function specialized for balancing energy consumption [28]. A relay selection scheme is proposed to maximize the lifetime of WBASNs through formulating and solving an optimization problem where relay selection of each node acts as optimization variable [29]. Traffic uncertainty causes degradation of network performance and interruption to operate protocols [30]. Therefore, design of network protocol should take into account some uncertain factors in order to make it realistic [31]. In the WBASN, the sensed data is generated by event-driven and this makes the algorithm to be outdated. For this reason, some optimization schemes are proposed to consider the event-driven data generation. A heuristic approach combining deterministic and probabilistic variable fixing strategies is proposed for WBASN optimal design, formulated as an integer programming problem [32]. An original optimization algorithm is proposed that exploits suitable linear relaxations to guide a randomized fixing of the variables [33]. It is supported by an exact large variable neighborhood search. Several robust algorithms considered the SINR. The robust optimization scheme using SINR is proposed to deal with the jamming problem in wireless networks [34]. The stochastic programming-based convex optimization with the probabilistic SINR constraints is proposed to optimize the transmission power [35]. A stochastic revenue optimization model based on bid pricing model for cellular networks is proposed [36]. There also exist techniques that integrate various functions of wireless networks. An integrated optimization is proposed to handle the collide functions and to allocate the radio resources [37]. An optimal cross layer design is developed in order to construct hierarchical system [38].

In conventional studies, the objective of transmission power control schemes is to minimize the energy consumption [21], or to maximize the data rate in considering interference occurred in inter-WBASN [7]. Actually, determining the data rate according to QoS requirements in wireless environment is one of the important issues rather than merely considering high transmission rates or energy efficiency [39]. However, as these schemes do not consider the QoS of each user in the WBASN environment, transmission power could operate regardless of the required data rate. Thus, there may be a limit in the ability to provide fair QoS to the users in WBASNs. In this paper, we propose an algorithm that can achieve fair QoS satisfaction across all users in WBASNs considering different QoS levels of the users. First of all, a utility function, which indicates how closely the current data rate matches the required data rate, is defined. Next, based on information from neighboring WBASNs, each network derives the transmission power needed for a QoS satisfaction value equal to the average QoS satisfaction value of the neighboring WBASNs. We use the Cucker–Smale model [40] in calculating the appropriate transmission power for each user. The Cucker–Smale model is typically used to simulate phenomena in which organisms move in groups, based on individual behavior with simple rules, using limited information. The model is thus suitable for the WBASN environment, where the transmission power must be controlled in a distributed manner, because it uses only information available from the neighboring nodes. In addition, the Cucker–Smale model can be applied to solving the problem of fair distribution of a satisfactory QoS in all neighboring WBASNs, because it synchronizes the specific variables of each entity to the same value.

## 3. System Model

**Figure 2:**
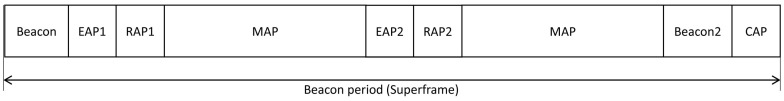
The layout of access phase in superframe. EAP, exclusive access phase; RAP, random access phase; MAP, managed access period; CAP, contention access period.

In this paper, we employ IEEE 802.15.6-based system as a target system [5]. In IEEE 802.15.6, multiple nodes belong to one coordinator (or hub). The hub shall operate in one of the three access modes: ‘beacon mode with superframe’, ‘non-beacon mode with superframe’, and ’non-beacon mode without superframe’. The superframe is divided into contention-based period and managed access period (MAP) as shown in Figure 2. In the contention-based period, there are three types of access phases: exclusive access phase (EAP), random access phase (RAP), and contention access period (CAP). In this period, sensor nodes use CSMA/CA or slotted ALOHA methods to access medium. Otherwise, in the MAP, it is possible to allocate resource by scheduling such as TDMA. If the beacon signal is available, the hub can determine whether to use each access phase or not, and broadcast some information such as the value of the utility function. For this reason, the beacon mode with superframe is used to facilitate a WBASN in our system.

**Figure 3:**
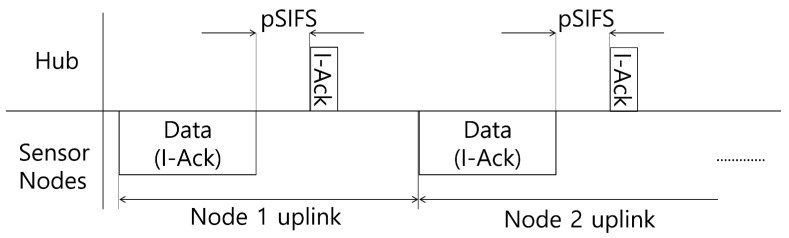
Example of scheduling in MAP. I-Ack, immediate-Ack. pSIFS, Short Inter-Frame Spacing.

A WBASN can consist of a hub and multiple sensor nodes. Each sensor node has a different QoS requirement, which leads to scheduling issue on resource allocation in the given MAC protocol. In this paper, we assumed that there is no intra-WBASN collision by assuming the TDMA-based WBASN protocol such as IEEE 802.15.6, in order to focus on the performance evaluation of our algorithm proposed in the context of control over inter-WBASN collision. The hub allocates time resources of the MAP to each sensor node by using the beacon signal. Figure 3 shows medium access in the MAP. Each sensor node periodically generates data and transmits it at the allocated time interval. At this time, the sensor node set the Ack field in the MAC header to immediate-Ack (I-Ack). After data transmission is completed, the hub sends back I-Ack including the information of calculated transmission power to the sensor node. The sensor node can adjust its power level by using the power information in the I-Ack.

**Figure 4:**
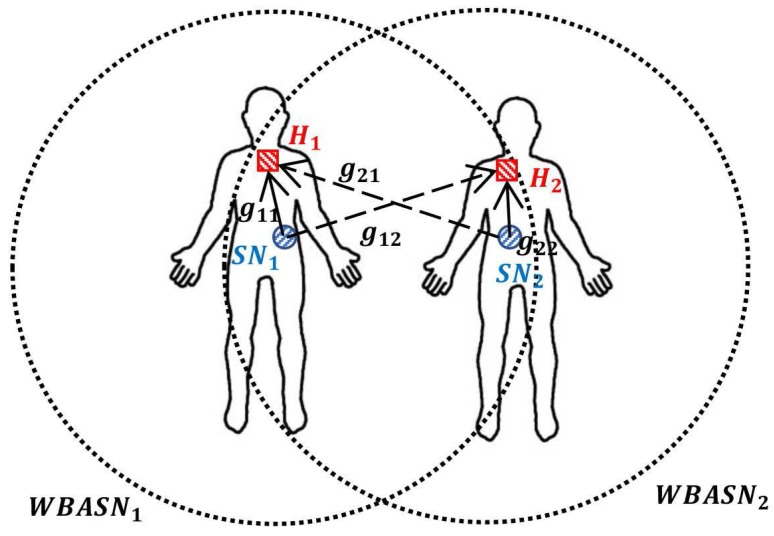
Inter-WBASN interference model.

It is assumed that a single sensor node $SN_{i}$ exists in $WBASN_{i}$ because contention-free MAC scheme is assumed to avoid intra-WBASN collision between sensor nodes associated with a WBASN, as shown in many studies on previous power control schemes in the WBASN environment [41,42,43,44]. As shown in Figure 4, there may be other WBASNs within transmission range, which may cause interference. Suppose that each user *i* constitutes a single WBASN $WBASN_{i}$ and one hub $H_{i}$ in $WBASN_{i}$ collects information from each sensor node. The coefficient $g_{ij}$ represents the channel gain between the transmitter *i* and the receiver *j*, i.e., The coefficient $g_{ij}$ represents intra-WBASN channel gain in the WBASN when i=j, and inter-WBASN interference gain between different WBASNs when i≠j. For example, in Figure 4, $SN_{1}$ tries to transmit measured data to $H_{1}$ in $WBASN_{1}$, which it belongs to, and, at the same time, $SN_{2}$ tries to transmit data to $H_{2}$ in $WBASN_{2}$, which causes interference to each other’s network.

The SINR, $\gamma_{i}\left(t\right)$, of the *i*-th WBASN at time *t* is:

(1) $$\gamma_{i}\left(t\right)=\frac{p_{i}\left(t\right)g_{ii}}{\sum_{j\in N_{i}:j\neq i}p_{j}\left(t\right)g_{ji}+n_{0}},\;i=1,2,\cdots ,N$$

where $p_{i}\left(t\right)$ is the transmission power of $SN_{i}$, $N_{i}$ is the set of neighboring nodes within transmission range of $SN_{i}$, and $n_{0}$ is additive white Gaussian noise (AWGN). According to Shannon’s theory, the data rate, $R_{i}\left(t\right)$, of the *i*-th WBASN at time *t* is described as follows:

(2) $$R_{i}\left(t\right)=B\log_{2}\left(1+\gamma_{i}\left(t\right)\right),\;b/s$$

where *B* is the bandwidth of the channel. Finally, the energy efficiency, $E_{i}\left(t\right)$, of the *i*-th WBASN at time *t* is defined as,

(3) $$E_{i}\left(t\right)=\frac{R_{i}\left(t\right)}{p_{i}\left(t\right)},\;b/J$$

## 4. Cucker–Smale Model

When observing naturally occurring phenomena, organisms often move in groups based on individual behavior with simple rules, using limited information. For example, when birds move in groups, they all move at the same speed. Cucker and Smale proposed a model (the Cucker–Smale model) to simulate this phenomenon, using the rule that each object adjusts its speed and direction individually, in consideration of the speed and direction of neighboring objects [40].

This mathematical model deals with the relationship of individuals that interact with each other. Each entity interacts with its neighbors, to adjust its velocity according to the weighted mean of the relative velocities of other neighbors in the group together with its own velocity. Assuming that there are *N* entities, the position of the *i*-th entity is defined as $x_{i}\left(t\right)$, and the velocity is defined as $v_{i}\left(t\right)$. Based on the above description, the Cucker–Smale model can be expressed as follows:

(4) $$\frac{dx_{i}\left(t\right)}{dt}=v_{i}\left(t\right)$$

(5) $$v_{i}\left(t+1\right)-v_{i}\left(t\right)=\frac{\lambda }{N}\sum_{j=1}^{N}\psi \left(\left|x_{j}-x_{i}\right|\right)\left(v_{j}\left(t\right)-v_{i}\left(t\right)\right)$$

In the above equation, i=1,⋯,N, t>0, and λ is a non-negative value indicating the coupling strength between individuals, which also refers to the learning weight. ψ(·) is a function expressing the communication range and weight between the affected entities. A typical ψ(·) function is given as follows:

(6) $$\psi_{1}\left(\left|x_{j}-x_{i}\right|\right)=1$$

(7) $$\psi_{2}\left(\left|x_{j}-x_{i}\right|\right)=1_{\left|x_{j}-x_{i}\right|\leq r}$$

(8) $$\psi_{3}\left(\left|x_{j}-x_{i}\right|\right)=\frac{1}{{\left(1+{\left|x_{j}-x_{i}\right|}^{2}\right)}^{\beta }}$$

In the above equation, *r* is a positive number, and β is a non-negative value. In this paper, we will use Equation (7) to express the communication range between entities. Furthermore, Cucker and Smale demonstrated that the velocities of all individuals converge, when ψ(·) is a non-negative and non-increasing function. Therefore, flocking phenomena satisfy the two following time-asymptotic convergence properties.

(9) $$\lim_{t\to \infty }\left|v_{i}\left(t\right)-v_{j}\left(t\right)\right|=0,\;for\;i\neq j$$

(10) $$\underset{0\leq t<\infty }{\sup}\left|x_{i}\left(t\right)-x_{j}\left(t\right)\right|<\infty ,\;for\;i\neq j$$

Equations (9) and (10) show that the difference in velocity between individuals converges to zero over time, and the distance between individuals does not diverge.

## 5. Proposed Algorithm

The Cucker–Smale model described in Section 3 is suitable for application to distributed resource allocation, because each node can achieve the global goal by acquiring and processing local information. In the Cucker–Smale model, the velocity vectors of each node are synchronized, as detailed in Equation (9). If the operational principle of the Cucker–Smale model is applied to the synchronization of each WBASN, the data rate of each node can be synchronized. However, different applications exist in the WBASN environment, where the difference in data rate varies, from several tens of kilobits per second, to ten megabits per second. Therefore, QoS fairness cannot be achieved for each node, if they share the same data rate. Moreover, if all users have the same data rate, some users may consume power unnecessarily in maintaining a data rate that is higher than required. To solve these problems, we propose a new power control algorithm for use in the WBASN environment based on the Cucker–Smale model. In the proposed algorithm, termed the flocking-based transmission power control with utility (FTPC-U) algorithm, the QoS of each user is defined as a utility function and the transmission power is adjusted to synchronize the value of the utility. This algorithm also increases the energy efficiency of the network, by preventing unnecessary energy consumption in transmission, and enables each user to be provided with a fair QoS.

### 5.1. Utility Function

**Figure 5:**
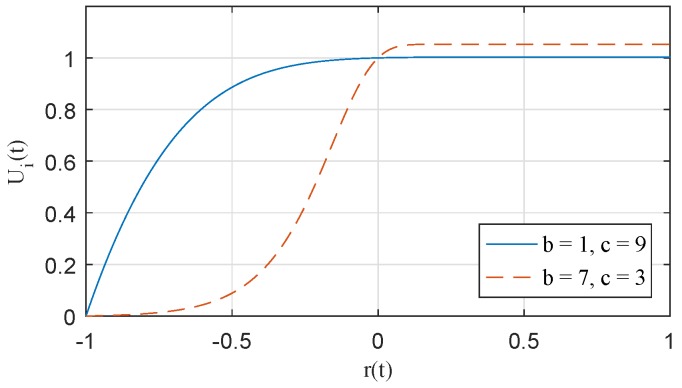
Proposed utility function.

In this paper, we define a utility function to express the QoS satisfaction of each user according to the data rate. Existing utility functions used to represent QoS satisfaction typically have a sigmoidal or logarithmic shape [45]. The shapes of these utility functions are applied depending on the type, and characteristics of the applications in use. We express two types of utility as one function. The utility, $U_{i}\left(t\right)$, of the *i*-th WBASN at time *t* is given as follows:

(11) $$U_{i}\left(t\right)=U_{max}-\frac{e^{-ce^{b\cdot r(t)}}}{e^{-ce^{-b}}-e^{-c}},\;r\left(t\right)=\frac{R_{i}\left(t\right)-R_{i}^{Req}}{R_{i}^{Req}}\left(-1\leq r(t)<\infty \right)$$

where *b* and *c* are the control parameters (b>0,c>0), and $R_{i}^{Req}$ is the required data rate of $WBASN_{i}$. The shape of the above function can be changed according to the values of control parameters, *b* and *c*, as shown in Figure 5. The term, $U_{max}=1+e^{-c}/\left(e^{-ce^{-b}}-e^{-c}\right)$, refers to the maximum possible value of the utility. In this paper, $R_{i}^{Req}$ does not change over time, but has a fixed value for each user. Since $R_{i}\left(t\right)$ has a minimum value of zero, r(t) is greater than or equal to −1. If $R_{i}\left(t\right)$ is zero, the value of r(t) is −1, and $U_{i}\left(t\right)$ is zero. If the values of $R_{i}\left(t\right)$ and $R_{i}^{Req}$ are same, the value of r(t) is zero, and the value of $U_{i}\left(t\right)$ is one. r(t) goes to infinity as $R_{i}\left(t\right)$ goes to infinity. The value of $U_{i}\left(t\right)$ is maximized in this case ($U_{i}\left(t\right)=U_{max}$).

### 5.2. Operation of the FTPC-U algorithm

We use the above utility function to calculate the utility value of each user, and apply it to the Cucker–Smale model to adjust the transmission power by synchronizing the value of utility function. The operational procedure of the proposed algorithm is as follows:

1. At time *t*, the sensor node $SN_{i}$ of each network $WBASN_{i}$ transmits a data packet to the hub $H_{i}$ with a transmission power given by $p_{i}\left(t\right)$.
2. $H_{i}$ measures the SINR $\gamma_{i}\left(t\right)$ based on the received packet.
3. $H_{i}$ computes the current data rate $R_{i}\left(t\right)$ using Equation (2).
4. $H_{i}$ calculates the current utility value $U_{i}\left(t\right)$ using Equation (11).
5. When the calculated utility is greater than one, i.e., $R_{i}\left(t\right)>R_{i}^{Req}$, $H_{i}$ reduces the transmission power of $SN_{i}$, such that the current data rate $R_{i}\left(t\right)$ becomes the required data rate $R_{i}^{Req}$. Using the SINR information at time *t*, $p_{i}^{Req}\left(t\right)$, the transmission power required to convert $R_{i}\left(t\right)$ to $R_{i}^{Req}$, can be obtained from Equations (1) and (2).
  (12) $$\begin{array}{cc} R_{i}^{Req} & =B\log_{2}\left(1+\gamma_{i}^{Req}\left(t\right)\right)\\ & =B\log_{2}\left(1+\frac{p_{i}^{Req}\left(t\right)g_{ii}}{\sum_{\forall j,j\neq i}^{N_{i}}p_{j}\left(t\right)g_{ji}+n_{0}}\right)\\ & =B\log_{2}\left(1+p_{i}^{Req}\left(t\right)\times \frac{\gamma_{i}\left(t\right)}{p_{i}\left(t\right)}\right) \end{array}$$
  where $\gamma_{i}^{Req}\left(t\right)$ is the SINR required for the hub $H_{i}$ to obtain the required data rate $R_{i}^{Req}$ considering the channel conditions and interference from neighboring WBASNs at time *t*. From Equation (12), we can determine $p_{i}^{Req}\left(t\right)$ as follows:
  (13) $$p_{i}^{Req}\left(t\right)=\max\left[p_{min},\min\left[p_{max},\left(2^{\frac{R_{i}^{Req}}{B}}-1\right)\times \frac{p_{i}\left(t\right)}{\gamma_{i}\left(t\right)}\right]\right]$$
  where $p_{max}$ and $p_{min}$ are the predetermined maximum and minimum transmission power, respectively.
6. $H_{i}$ transmits information of $U_{i}\left(t\right)$ to neighboring WBASNs.
7. $H_{i}$ computes the next target data rate using Equation (14) (defined below), with the received $U_{i}\left(t\right)$ information and the Cucker–Smale model, as follows:
  (14) $$U_{i}\left(t+1\right)-U_{i}\left(t\right)=\frac{\lambda }{N}\sum_{j=1}^{N}\psi \left(\left|x_{j}-x_{i}\right|\right)\cdot \left(U_{j}\left(t\right)-U_{i}\left(t\right)\right)$$
  If any WBASN is out of communication range, $\psi (\left|\cdot \right|)=0$. Assuming $\psi \left(\left|x_{j}-x_{i}\right|\right)=1_{\left|x_{j}-x_{i}\right|\leq r}$ and λ=1, Equation (14) can be expressed simply, as the following equation:
  (15) $$U_{i}\left(t+1\right)=\frac{1}{\left|N_{i}\right|}\sum_{j\in N_{i}}U_{j}\left(t\right)$$
  where $N_{i}$ is a set of WBASNs neighboring $H_{i}$, and $\left|\cdot \right|$ denotes the cardinality of the corresponding set.
8. If the difference between $U_{i}\left(t+1\right)$ and $U_{i}\left(t\right)$ is very small, i.e., $\left|U_{i}\left(t+1\right)-U_{i}\left(t\right)\right|<\epsilon$, where ϵ is a very small predefined constant, $H_{i}$ determines that the utility has converged and does not adjust the transmission power.
9. $H_{i}$ determines the transmission power to be used at the next time interval $p_{i}\left(t+1\right)$ based on the calculated target utility $U_{i}\left(t+1\right)$. $p_{i}\left(t+1\right)$ can be expressed as a single Equation (16) based on Equations (1), (2), and (11), with the same method used to define Equation (13).
  (16) $$p_{i}\left(t+1\right)=\max\left\{p_{min},\min\left\{p_{max},\frac{p_{i}\left(t\right)}{\gamma_{i}\left(t\right)}\times \left(2^{\frac{R_{i}^{Req}\ln{\left(-\frac{1}{c}\ln\left(\alpha +e^{-c}-\alpha U_{i}\left(t+1\right)\right)\right)}^{-b}+R_{i}^{Req}}{B}}-1\right)\right\}\right\}$$
  where $\alpha =e^{-ce^{-b}}-e^{-c}$.

**Figure 6:**
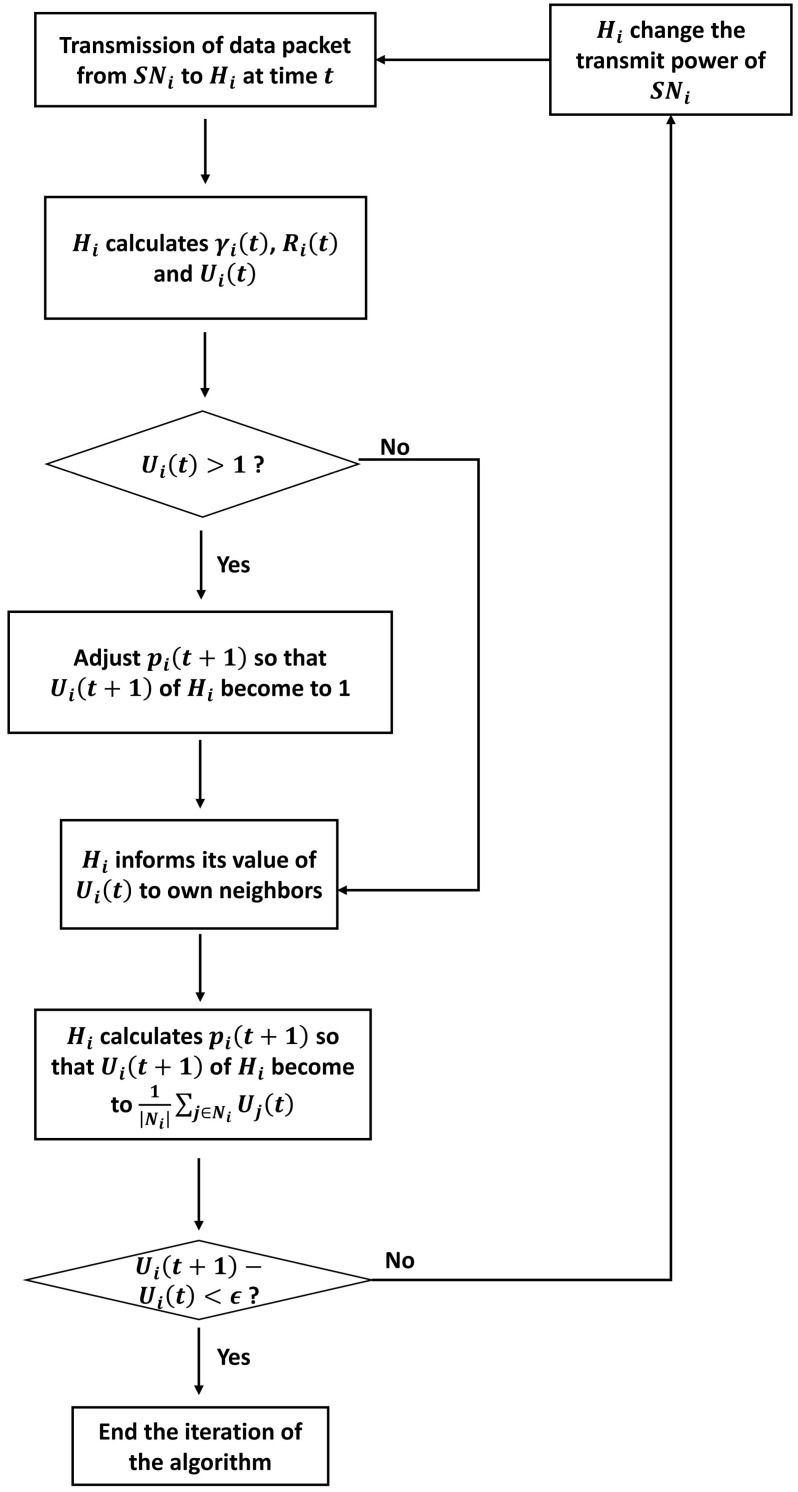
Operational procedure of the proposed algorithm.

Algorithm 1 is a pseudo-code of the FTPC-U algorithm. During the execution of the proposed algorithm, it is possible to prevent sensor nodes from wasting transmission power in each WBASN, using Equation (13). It is also possible to synchronize the utilities of the WBASNs dispersively, using Equation (16). The data rate of the coordinator $H_{i}$ which is dependent on the user *i* is related to the transmission power of all other neighboring sensor nodes $SN_{j}$ ($\forall j\in N_{i},j\neq i$). Accordingly, adjusting the transmission power of one sensor node affects the data rate of all other coordinators. Therefore, the operating procedure of the proposed algorithm must be repeated, such that the utility value converges completely. Figure 6 is a schematic diagram of the operational procedure detailed above. Table 1 shows a comparison between elements used in the Cucker–Smale model and the proposed algorithm.
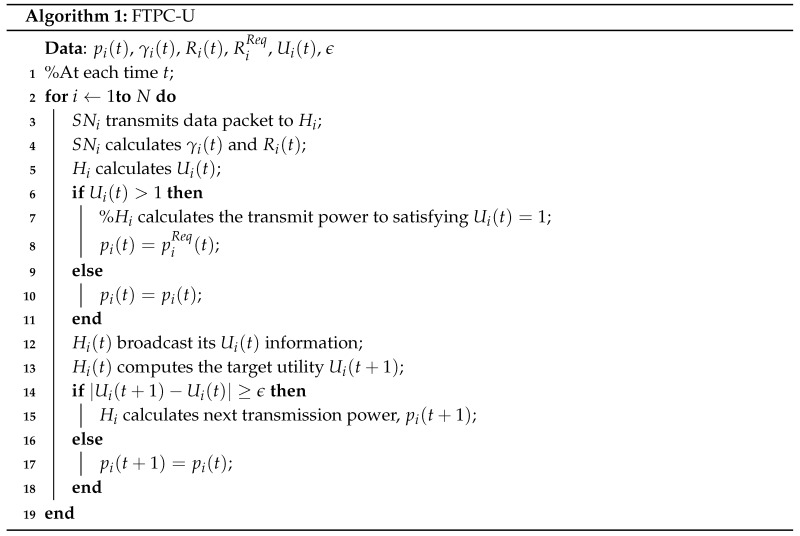


### 5.3. Convergence of the FTPC-U Algorithm

In this section, to show the stability of the proposed algorithm, we prove its convergence. First, we consider Equation (14) in the continuous time domain, using the following equation:

(17) $$U_{i}\left(t+1\right)-U_{i}\left(t\right)=\frac{dU_{i}\left(t\right)}{dt}=\frac{\lambda }{N}\sum_{j=1}^{N}\psi \left(\left|x_{j}-x_{i}\right|\right)\cdot \left(U_{j}\left(t\right)-U_{i}\left(t\right)\right)$$

The center of $U_{i}\left(t\right)$, $U_{c}\left(t\right)$, is given by:

(18) $$U_{c}\left(t\right):=\frac{1}{N}\sum_{i=1}^{N}U_{i}\left(t\right)$$

By differentiating Equation (18), we obtain the following:

(19) $$\begin{array}{cc} \frac{dU_{c}\left(t\right)}{dt} & =\frac{1}{N}\sum_{i=1}^{N}\frac{dU_{i}\left(t\right)}{dt}\\ & =\frac{1}{N}\sum_{i=1}^{N}\sum_{j=1}^{N}\frac{\lambda }{N}\psi \left(\left|x_{j}-x_{i}\right|\right)\cdot \left(U_{j}\left(t\right)-U_{i}\left(t\right)\right)\\ & =\frac{\lambda }{N^{2}}\sum_{i=1}^{N}\sum_{j=1}^{N}\psi \left(\left|x_{j}-x_{i}\right|\right)\cdot \left(U_{j}\left(t\right)-U_{i}\left(t\right)\right)\\ & =\frac{\lambda }{2N^{2}}\sum_{i=1}^{N}\sum_{j=1}^{N}\psi \left(\left|x_{j}-x_{i}\right|\right)\cdot \left(U_{j}\left(t\right)-U_{j}\left(t\right)+U_{i}\left(t\right)-U_{i}\left(t\right)\right)\\ & =0 \end{array}$$

The derivative value in Equation (19) becomes zero, indicating that $U_{c}\left(t\right)=U_{c}\left(0\right)$. ${\hat{U}}_{i}\left(t\right)$, the fluctuations around $U_{c}\left(0\right)$, can be defined as follows:

(20) $${\hat{U}}_{i}\left(t\right):=U_{i}\left(t\right)-U_{c}\left(0\right)$$

From Equations (18) and (20), we have:

(21) $$\sum_{i=1}^{N}{\hat{U}}_{i}\left(t\right)=0$$

Here, we define U(t) as $U\left(t\right):=\{{\hat{U}}_{1}\left(t\right),{\hat{U}}_{2}\left(t\right),\cdots ,{\hat{U}}_{N}\left(t\right)\}\in R^{N}$, which is the set of ${\hat{U}}_{i}\left(t\right)$. The $L^{2}$-norm of this set, which denotes quantities proportional to the standard deviations of U(t), is given by:

(22) $$\left|\left|U(t)\right|\right|={\left(\sum_{i=1}^{N}{\left|{\hat{U}}_{i}\left(t\right)\right|}^{2}\right)}^{\frac{1}{2}}$$

Substituting Equation (20) into Equation (17) yields:

(23) $$\begin{array}{cc} \frac{d{\left|{\hat{U}}_{i}\left(t\right)\right|}^{2}}{dt} & =2{\hat{U}}_{i}\left(t\right)\cdot \frac{d\left|{\hat{U}}_{i}\left(t\right)\right|}{dt}\\ & =2{\hat{U}}_{i}\left(t\right)\cdot \frac{\lambda }{N}\sum_{j=1}^{N}\psi \left(\left|x_{j}-x_{i}\right|\right)\cdot ({\hat{U}}_{j}\left(t\right)-{\hat{U}}_{i}\left(t\right)) \end{array}$$

We calculate the derivative of ${\left|\left|U(t)\right|\right|}^{2}$ as follows:

(24) $$\begin{array}{cc} \frac{d{\left|\left|U(t)\right|\right|}^{2}}{dt} & =\frac{d}{dt}\sum_{i=1}^{N}{\left|{\hat{U}}_{i}\left(t\right)\right|}^{2}=\sum_{i=1}^{N}\frac{d{\left|{\hat{U}}_{i}\left(t\right)\right|}^{2}}{dt}\\ & =\frac{\lambda }{N}\sum_{i=1}^{N}2{\hat{U}}_{i}\left(t\right)\sum_{j=1}^{N}\psi (\left|x_{j}-x_{i}\right|)\cdot ({\hat{U}}_{j}\left(t\right)-{\hat{U}}_{i}\left(t\right))\\ & =-\frac{\lambda }{N}\sum_{i=1}^{N}\sum_{j=1}^{N}\psi \left(\left|x_{j}-x_{i}\right|\right)\cdot (2{\hat{U}}_{j}{\left(t\right)}^{2}-2{\hat{U}}_{j}\left(t\right){\hat{U}}_{i}\left(t\right))\\ & =-\frac{\lambda }{N}\sum_{i=1}^{N}\sum_{j=1}^{N}\psi \left(\left|x_{j}-x_{i}\right|\right)\cdot {\left|{\hat{U}}_{j}\left(t\right)-{\hat{U}}_{i}\left(t\right)\right|}^{2} \end{array}$$

We assume that the maximum difference in location between any two WBASNs in the network is given by $x_{max}=\max_{i,j}\left|x_{j}-x_{i}\right|$. Because $\psi \left(\cdot \right)$ is a non-negative and non-increasing function, we can rewrite Equation (24) as,

(25) $$\begin{array}{cc} \frac{d{\left|\left|U(t)\right|\right|}^{2}}{dt} & \leq -\frac{\lambda }{N}\sum_{i=1}^{N}\sum_{j=1}^{N}\psi \left(x_{max}\right)\cdot {\left|{\hat{U}}_{j}\left(t\right)-{\hat{U}}_{i}\left(t\right)\right|}^{2}\\ & =-\frac{\lambda }{N}\psi \left(x_{max}\right)\sum_{i=1}^{N}\sum_{j=1}^{N}\left({\hat{U}}_{j}{\left(t\right)}^{2}+{\hat{U}}_{i}{\left(t\right)}^{2}-2{\hat{U}}_{j}\left(t\right){\hat{U}}_{i}\left(t\right)\right)\\ & =-\frac{\lambda }{N}\psi \left(x_{max}\right)\left(\sum_{i=1}^{N}\sum_{j=1}^{N}2{\hat{U}}_{i}{\left(t\right)}^{2}-2\sum_{i=1}^{N}{\hat{U}}_{i}\left(t\right)\sum_{j=1}^{N}{\hat{U}}_{j}\left(t\right)\right)\\ & =-\frac{\lambda }{N}\psi \left(x_{max}\right)\left(2N{\left|\left|U(t)\right|\right|}^{2}-0\right)\\ & =-2\lambda \psi \left(x_{max}\right){\left|\left|U(t)\right|\right|}^{2} \end{array}$$

By solving Equation (25), we can get the following inequalities:

(26) $${\left|\left|U(t)\right|\right|}^{2}\leq {\left|\left|U(0)\right|\right|}^{2}e^{-2\lambda \psi \left(x_{max}\right)t}$$

(27) $$\left|\left|U(t)\right|\right|\leq \left|\left|U(0)\right|\right|e^{-\lambda \psi \left(x_{max}\right)t}$$

which dictate that the proposed algorithm achieves exponential convergence.

## 6. Simulation Results

**Figure 7:**
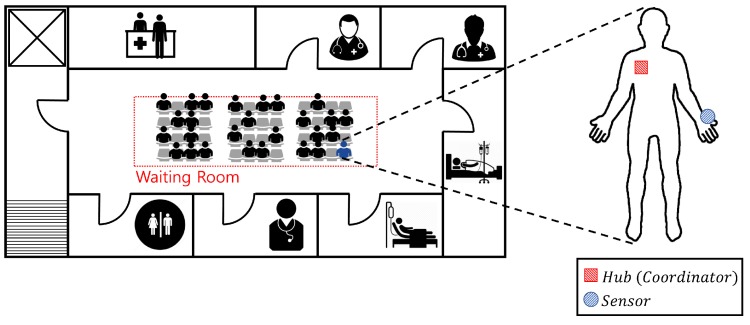
Network environment, assuming a hospital waiting room.

In this section, we evaluate the performance of the proposed algorithm using Monte-Carlo simulations. We assume the network topology proposed in Section 2. Each WBASN operates in beacon mode according to the IEEE 802.15.6 standard, and exchanges utility information. A hospital waiting room was considered, as a dense environment where interference could occur. Figure 7 depicts the network environment, assuming a hospital waiting room. The area is set to 14 m × 4 m, where the maximum number of WBASNs is 48. In all subsequent simulations, we will use a logarithmically-shaped utility function, with control parameters b=1 and c=9. Table 2 summarizes the applications used in the simulations detailed in this paper, and considers the requirements for various services used in the WBASN [1,46,47].

The simulation considers an uplink situation where measured data is transmitted from the sensor node to the coordinator. The channel gain in decibels is given by $g_{ij}=16.7\log_{10}d-0.45$, where *d* is the distance [48]. Table 3 summarizes the commonly-used parameters in this paper.

Furthermore, we compared the performance of the proposed algorithm with two algorithms: PAPU and transmission power control for body area network (TPC-BAN). PAPU controls transmission power based on game theory and aims to increase the transmission rate within a given power limit, and calculates the Nash equilibrium using the best response concept to determine the optimized transmission power value by distributed processing [7]. This algorithm is adapted to update the transmit power according to the occurrence of the event. There are two kinds of events. The first one occurs when the transmission power is adjusted in the other network, and the second event occurs when the SINR variation according to the time is equal to or greater than a predetermined threshold value. When an event occurs, the hub changes the transmit power according to the best response and informs the neighboring network of the change of the transmit power of the hub by broadcasting. TPC-BAN controls transmission power using the detected RSSI [23]. In TPC-BAN, the hub updates its own average RSSI by giving weighted parameter in order to mitigate the effects of instantaneous channel mutation. The hub determines whether to control transmission power or not by comparing the average RSSI value with the predetermined target reception power. If the average RSSI value is located in the offset range of the target RSSI, the hub does not change the power of sensor node. However, if the average RSSI value is out of the offset range of the target RSSI, the hub changes the transmission power of the sensor node. The algorithm periodically updates its average RSSI and adjusts the transmit power of the sensor nodes until it reaches the offset range of the target RSSI.

**Figure 8:**
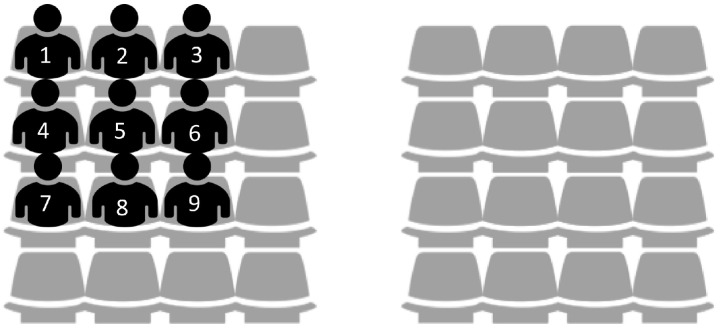
Location distribution of each WBASN in the performance-evaluation-over-time.

To verify the performance of the proposed algorithm, we create a network environment where nine WBASNs are placed close together, at specific locations, as shown in Figure 8. Table 4 shows information of applications used by each WBASN in this environment.

**Figure 9:**
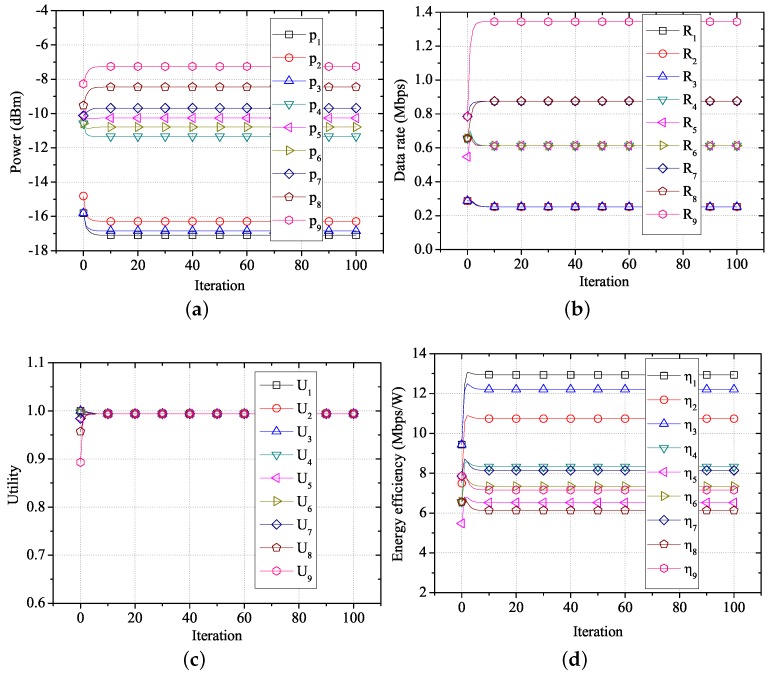
Performance of FTPC-U over time. (**a**) Transmission power over time; (**b**) data rate over time; (**c**) utility over time; (**d**) energy efficiency over time.

Figure 9 shows the changes in performance for each user over time, using the proposed algorithm in the network environment shown in Figure 8 and Table 4. As shown in Figure 9a, $WBASN_{1}$, $WBASN_{2}$, and $WBASN_{3}$ have a low required data rate (288 kbps). Thus, each coordinator reduces the transmission power of the corresponding sensor nodes, which do not require a large transmission power. In the case of $WBASN_{9}$, the coordinator increases the transmission power of the sensor node, which has a high required data rate (1.536 Mbps). By adjusting the transmission power, the data rate of each WBASN converges to a specific value, based on the required data rate, as shown in Figure 9b. As shown in Figure 9c, the utility of each user converges to the same value. As shown in Figure 9d, when the proposed algorithm is applied, only the transmission power needed for data transfer at the required data rate is consumed. Hence, it shows that transmission using the proposed algorithm has a high energy-efficiency.

**Figure 10:**
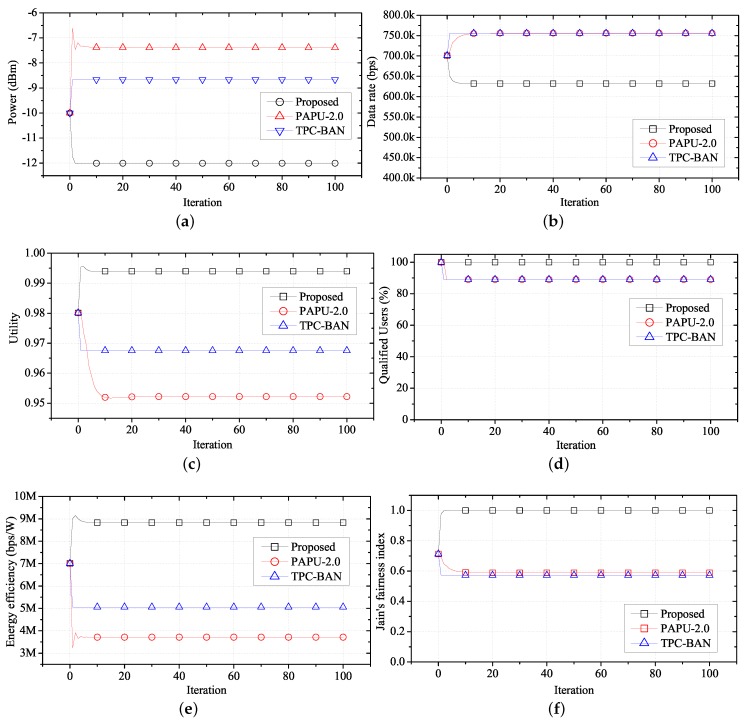
Performance of FTPC-U compared with PAPU and TPC-BAN over time. (**a**) Change in average transmit power over time; (**b**) change in average data rate over time; (**c**) change in average utility over time; (**d**) change in qualified users over time; (**e**) change in average energy efficiency over time; (**f**) change in fairness over time. PAPU, proactive power update; TPC-BAN, transmission power control for body area network.

Figure 10 compares the performance of the proposed algorithm with PAPU and TPC-BAN. With the distribution of locations and applications as shown in Figure 8 and Table 4, we conduct simulations to obtain an average performance value of each WBASN over time when the proposed algorithm, PAPU, and TPC-BAN are employed. The purposes of PAPU and TPC-BAN are to increase the overall data rate of the entire node and to satisfy global target RSSI value, respectively, while the proposed scheme aims to increase the fairness of the QoS, and decrease the transmission power of each user, simultaneously. Therefore, when using the proposed scheme, lower transmission powers and average data rates are observed as shown in Figure 10a,b, and a higher utility value is observed as shown in Figure 10c. Since with the proposed algorithm each user has a high average utility, the number of users with a satisfactory QoS is greater than when PAPU or TPC-BAN is used, as shown in Figure 10d. In addition, since the proposed scheme requires a lower transmission power on average, energy efficiency is high, as shown in Figure 10e.

To evaluate the QoS fairness of network users, we use Jain’s fairness index [49], defined as follows:

(28) $$J\left(x_{1},x_{2},\cdots ,x_{N}\right)=\frac{{\left(\sum_{i=1}^{N}x_{i}\right)}^{2}}{N\sum_{i=1}^{N}x_{i}^{2}}$$

where $x_{i}=R_{i}\left(t\right)/R_{i}^{Req}$. Jain’s fairness index defines a variable $x_{i}$ that indicates how close the current data rate $R_{i}\left(t\right)$ is to the required data rate $R_{i}^{Req}$ for each of the *N* users in the network. As the difference in $x_{i}$ is reduced, the QoS for each user is satisfied fairly, and the value of Jain’s fairness index approaches one. Figure 10f is a graph comparing the Jain’s fairness index of the proposed method with the index obtained using PAPU and TPC-BAN. Since the proposed algorithm synchronizes the utility, higher QoS fairness is observed than with PAPU and TPC-BAN.

**Figure 11:**
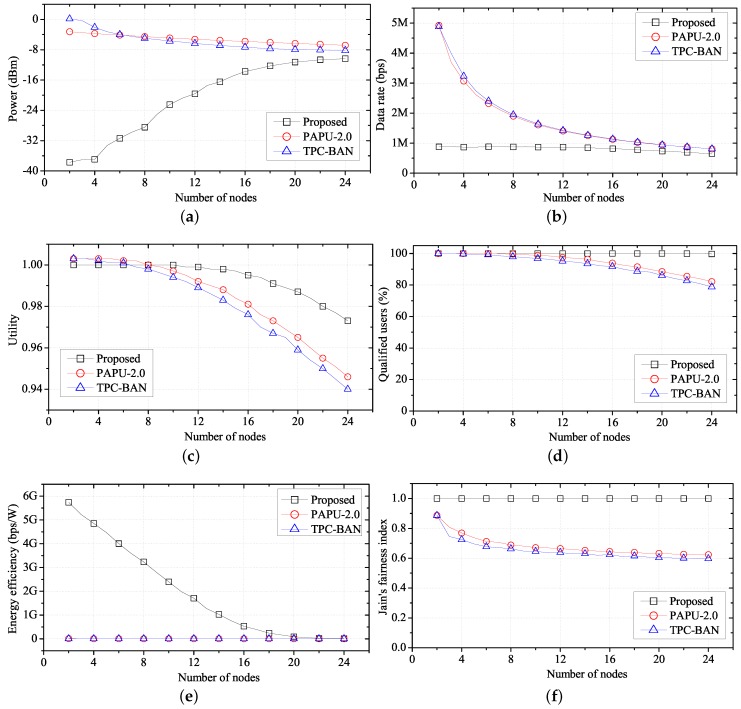
Performance of FTPC-U compared with PAPU and TPC-BAN according to the number of nodes. (**a**) Change in average transmit power; (**b**) change in average data rate; (**c**) change in average utility; (**d**) change in qualified users; (**e**) change in average energy efficiency; (**f**) change in fairness.

Figure 11 shows the average performance of FTPC-U, PAPU, and TPC-BAN according to the number of nodes. We change the number of nodes from 2–24, placing each node in a 14 m × 4 m space, as shown in Figure 7. Each node is randomly assigned one of the applications listed in Table 2. Figure 11a shows the transmission power according to the number of nodes. In this case, the overall interference increases as the number of nodes increases. With PAPU and TPC-BAN, the hub tries to reduce the transmission power of the sensor node as the interference increases. However, with FTPC-U, the hub adjusts the transmission power of each sensor node using Equation (16), for synchronization of the utilities. In this case, if a decrease in SINR caused by the increase in interference from Equation (16) occurs, the transmission power of the sensor node is increased. Figure 11b is a graph showing the data rate according to the number of nodes. When the number of nodes is small, we observe a high data rate, which decreases as the number of nodes increases, using PAPU or TPC-BAN. In contrast, with FTPC-U, we observe a low data rate when the number of nodes is small, because the required data rate can be achieved without a high transmission power. We note that Figure 11c shows an average utility of one, until the number of nodes is about 12. This shows that the actual data rate is close to the required data rate. Therefore, even though the data rate observed in Figure 11b is low, the user does not perceive a deterioration in performance. We note from Figure 11c that the effect of the number of nodes on performance is less prevalent using FTPC-U, which controls the transmission power considering the interference caused by the increase in the number of nodes, and the data rate of each user, than it is with PAPU or TPC-BAN. Therefore, as shown in Figure 11d, the number of users with a satisfactory QoS is kept high with FTPC-U, but decreases gradually with PAPU and TPC-BAN. Since the minimum power required for a satisfactory QoS is used with the proposed algorithm, the energy efficiency is high as shown in Figure 11e. Figure 11f shows the QoS fairness according to the number of nodes. FTPC-U shows higher QoS fairness than PAPU and TPC-BAN because it adapts the synchronization of utilities.

**Figure 12:**
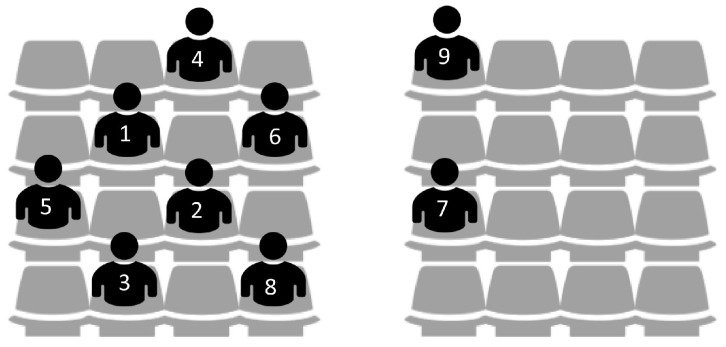
Location distribution of each WBASN in the performance-evaluation-with-entry model.

Figure 12 shows the order that each WBASN arrives to the network environment, and its corresponding location information. In the initial stage, we assume that only four WBASNs, $WBASN_{1}$–$WBASN_{4}$, exist. Then, one WBASN sequentially enters when the number of iterations increases by a 100, so that up to nine WBASNs are in the network environment. To evaluate performance in dense situations, an arriving WBASN is placed in a location adjacent to an existing WBASN. Table 5 shows the application information of each WBASN in this environment.

**Figure 13:**
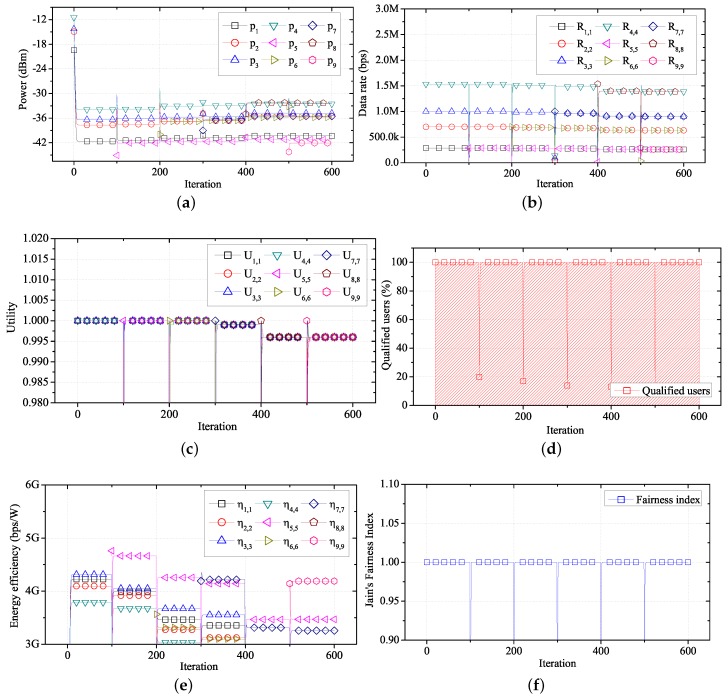
Performance of FTPC-U over time in the entry model. (**a**) Change in transmit power over time in the entry model; (**b**) change in data rate over time in the entry model; (**c**) change in utility over time in the entry model; (**d**) change in qualified users over time in the entry model; (**e**) change in energy efficiency over time in the entry model; (**f**) change in fairness over time in the entry model.

Figure 13 shows the change in performance according to the entry of WBASNs, when the network environment detailed in Figure 12 and Table 5 is applied. Figure 13a,b shows the changes in transmission power and data rate when a new WBASN enters an existing network environment. Here, when the new WBASN enters, a temporary change in interference may cause a temporary change to the transmission power, and a decrease in the data rate. In addition, the utility of each WBASN can be reduced temporarily, as shown in Figure 13c. Due to the instantaneous change in utility, the number of users with a satisfactory QoS temporarily decreases, as shown in Figure 13d. As shown in Figure 13e, high energy efficiency is observed using the proposed algorithm, because only as much transmission power as required is consumed. Figure 13f shows the QoS fairness in the entry environment, indicating that the proposed scheme achieves high QoS fairness through utility synchronization.

**Figure 14:**
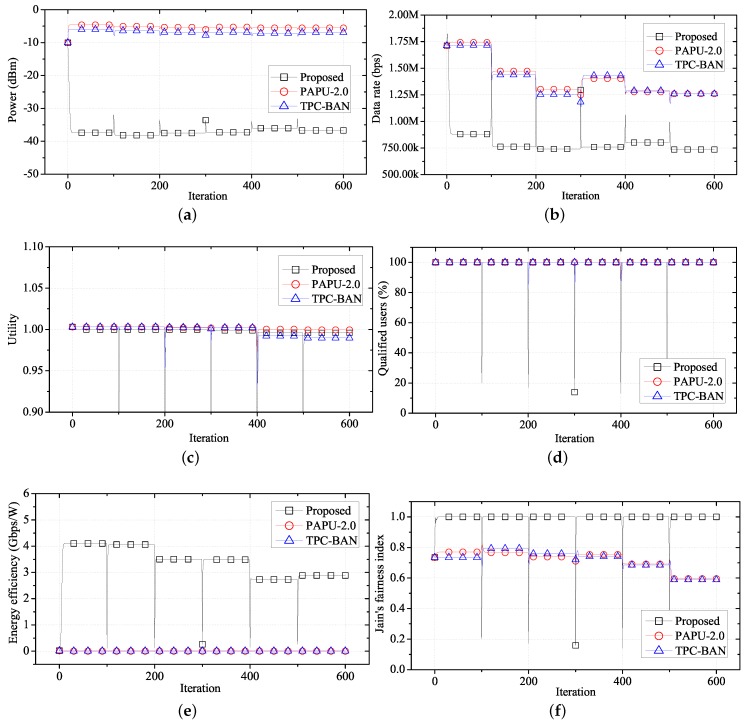
Performance of FTPC-U compared with PAPU and TPC-BAN over time in the entry environment. (**a**) Change in average transmit power; (**b**) change in average data rate; (**c**) change in average utility; (**d**) change in qualified users; (**e**) change in average energy efficiency; (**f**) change in fairness.

Figure 14 shows the average performance of FTPC-U, PAPU, and TPC-BAN when a new WBASN enters an existing network environment. With the entry model and applications as shown in Figure 12 and Table 5, we evaluate the average performance value of each WBASN with FTPC-U, PAPU, and TPC-BAN. As shown in Figure 14a, transmission power is not consumed unnecessarily with FTPC-U, as the influence of interference and required data rate are considered, whereas PAPU and TPC-BAN controls the transmission power to maximize the data rate and to satisfy global target RSSI, respectively. Therefore, as shown in Figure 14c, although a lower data rate is observed using FTPC-U than with PAPU or TPC-BAN, the converged utility value is almost the same in both cases. Moreover, since all of algorithms maintain a utility close to one, the number of users with a satisfactory QoS is also maintained at about 100% as shown in Figure 14d. Figure 14e shows the energy efficiency in the WBASN entry environment. FTPC-U has a significantly higher energy efficiency than PAPU or TPC-BAN, because it operates at low power. Figure 14f shows the QoS fairness for users in the WBASN entry environment. When PAPU or TPC-BAN is used, the QoS fairness of the users is reduced each time a new WBASN enters the network environment. However, if FTPC-U is used, Jain’s fairness index is maintained at about one. In the entry environment, the performance temporarily decreases, because interference increases at the moment of entry. The overall performance degradation at this moment is larger with FTPC-U than with PAPU or TPC-BAN. This is because, with PAPU or TPC-BAN, the transmission power of most of the sensor nodes is adjusted to a relatively high level, however, with FTPC-U, many sensor nodes are adjusted to use a low transmission power, and sensor nodes with low transmission powers are more seriously affected by interference caused by a newly entering WBASN. Nevertheless, using FTPC-U, we observe high performance in the converged state.

## 7. Conclusions

We introduced FTPC-U, which is a flocking-inspired algorithm for controlling transmission power using the Cucker–Smale model, to solve interference problems that occur in the WBASN environment and to guarantee QoS fairness between users. With the proposed algorithm, a fair QoS is guaranteed for each user, by calculating a utility that expresses the QoS satisfaction numerically and synchronizing this utility with those of neighboring WBASNs. Results of simulations show that FTPC-U operates stably in both static and dynamic states. Comparing to PAPU and TPC-BAN, FTPC-U consumes less power and exhibits higher or equal utility values. Improved energy efficiency and QoS fairness were observed with FTPC-U, in both static and entry environments, compared with PAPU and TPC-BAN. As a result, FTPC-U can effectively mitigate interference in dense environments and ensure that the QoS satisfaction value for each user is fair. In addition, we expect that using this algorithm, high performance can be achieved with other types of networks where various applications coexist in a dense and highly mobile environment.

In this paper, we assumed that only one sensor node is associated with a WBASN. However, it is more natural to assume the coexistence of multiple sensor nodes associated with a WBASN. Each sensor node associated with a WBASN may have a different QoS requirement, and this leads to the scheduling issue in a WBASN. For this reason, it is necessary to consider QoS-fairness of multiple sensor nodes in a WBASN. Therefore, a study on the joint transmission power control and resource allocation algorithm for QoS-fairness of all users considering both intra-WBASN and inter-WBASN collisions would be our further work. In addition, the proposed algorithm assumes that all hubs and sensor nodes operate normally without any malicious node. However, the performance of the network may deteriorate by malicious nodes, which may cause interference increase, data collision and obstruction of convergence. We have a plan to extend the proposed algorithm considering the existence of malicious nodes.

## Figures and Tables

**Table 1:** Comparison between elements used in the Cucker–Smale model and the proposed algorithm. FTPC-U, locking-based transmission power control with utility.

Elements	Cucker–Smale Model	FTPC-U
Adjusted parameter	Velocity	Transmission power
Communication range function	$\psi \left(\left|\cdot \right|\right)$	$1_{\left|x_{j}-x_{i}\right|\leq r}$
Comparison of algorithm model	$\frac{dv_{i}\left(t\right)}{dt}=\frac{\lambda }{N}\sum_{j=1}^{N}\psi (\left|x_{j}-x_{i}\right|)(v_{j}\left(t\right)-v_{i}\left(t\right))$	$\frac{dU_{i}\left(t\right)}{dt}=\frac{1}{\left|N_{i}\right|}\sum_{j\in N_{i}}U_{j}\left(t\right)$
Objective to converge	Velocity	Utility

**Table 2:** Type of application used in the WBASN and required data rate.

Application Name	Required Data Rate
ECG	288 kbps
Artificial retina	700 kbps
Capsule endoscope	1 Mbps
EMG	1.536 Mbps

**Table 3:** Commonly-used simulation parameters.

Parameter	Value
Network size	14 m × 14 m
Size of each seat	1 m × 1 m
Location of hub	Center of assigned seat
Location of sensor node	Uniformly random distribution within the area of each seat
Initial transmission power	Uniformly random allocation in (−14,−6), dBm
Bandwidth	1 MHz
Path loss model	$g_{ij}=16.7\log_{10}d-0.45$ (in dB, *d* = distance)
Convergence error (ϵ)	$10^{-4}$

**Table 4:** Applications used by WBASNs in the performance-evaluation-over–time.

WBASN ID	Application	WBASN ID	Application
1	ECG (288 kbps)	6	Artificial retina
2	ECG	7	Capsule endoscope (1 Mbps)
3	ECG	8	Capsule endoscope
4	Artificial retina (700 kbps)	9	EMG (1.536 Mbps)
5	Artificial retina	-	-

**Table 5:** Applications used by WBASNs in the performance-evaluation-with-entry model.

WBASN ID	Application	WBASN ID	Application
1	ECG (288 kbps)	6	Artificial retina
2	Artificial retina (700 kbps)	7	Capsule endoscope (1 Mbps)
3	Capsule endoscope (1 Mbps)	8	EMG
4	EMG (1.536 Mbps)	9	ECG
5	ECG	-	-

## Supplementary Materials

Supplementary File 1
