# Supplementary material for: Bio-Inspired Distributed Transmission Power Control Considering QoS Fairness in Wireless Body Area Sensor Networks
Source: Sensors (Basel). 2017 Oct 14;17(10):2344. doi: 10.3390/s17102344 (PMC5676673; doi:10.3390/s17102344)
Supplement: Supplementary File 1 [file sensors-17-02344-s001.docx]

**[ List of Modifications ]**

Dear, the Production Team.

Thank you for your kind instructions. Our final manuscript was modified, however, we had a problem with generate a pdf file although all of packages are installed well. Therefore, there may be some errors in pdf file. Please understand that we failed to prepare correct pdf file.

List of Modifications are as follows:

Line 114, 119 – We did not modify the name 'Chan-Jae Lee'. However, we find the author name on the MDPI homepage is written in 'Chan-jae Lee'. We wonder if you could change the author name on MDPI homepage to 'Chan-Jae Lee'?

Line 123 – Address was modified. (School of Electrical and Electronics Engineering, …)

Line 199 – The space between number and ‘mW’ was added in Figure 1.

Line 251 – pSIFS was defined in Figure 2.

Line 275, 280 – The square brackets are eliminated.

Line 345 – We added space before and after ‘=’ in Figure 5.

Line 588 – We changed square brackets to round brackets according to MDPI’s rule in Table 3.

Line 643 – We modified caption of Figure 9 and labels in each graph in this figure.

Modified caption: Performance of FTPC-U over time.

Line 657 - We modified caption of Figure 10 and labels in each graph in this figure.

Modified caption: Performance of FTPC-U compared with PAPU and TPC-BAN over time.

Line 677 - We modified caption of Figure 11 and labels in each graph in this figure.

Modified caption: Performance of FTPC-U compared with PAPU and TPC-BAN according to the number of nodes.

Line 718 - We modified caption of Figure 13 and labels in each graph in this figure.

Modified caption: Performance of FTPC-U compared with PAPU and TPC-BAN over time in the entry environment.

Line 737 - We modified caption of Figure 14 and labels in each graph in this figure.

Modified caption: Performance of FTPC-U over time

Line 755 – We wrote author contributions as follows:

Chan-Jae Lee contributed the paper by deriving initial results of simulation and by making the draft version of the paper. Ji-Young Jung contributed the paper by correcting some errata in the draft version of the paper, deriving results of simulation using TPC-BAN to clarify practical effect of the proposed algorithm, and writing the response letters. Jung-Ryun Lee was responsible for the main idea, theoretical analysis, coordination and proof reading of the paper.

Line 826, 957, 963, and 967 – We wrote the accessed date to each reference.
